# Elective cesarean section or not? Maternal age and risk of adverse outcomes at term: a population-based registry study of low-risk primiparous women

**DOI:** 10.1186/s12884-016-1028-3

**Published:** 2016-08-17

**Authors:** Lina Herstad, Kari Klungsøyr, Rolv Skjærven, Tom Tanbo, Lisa Forsén, Thomas Åbyholm, Siri Vangen

**Affiliations:** 1 Norwegian National Advisory Unit on Women’s Health, Women and Children’s Division, Oslo University Hospital Rikshospitalet, PO Box 4950, Nydalen N-0424 Oslo, Norway; 2 Medical Birth Registry of Norway, Norwegian Institute of Public Health, Bergen, Norway; 3 Departments of Global Public Health and Primary Care, University of Bergen, Bergen, Norway; 4 University of Oslo, Oslo, Norway; 5 Department of Gynecology, Oslo University Hospital, Oslo, Norway; 6 Norwegian Institute of Public Health, Oslo, Norway; 7 Department of Obstetrics, Oslo University Hospital, Oslo, Norway

**Keywords:** Maternal age, Low-risk population, Delivery, Obstetric, Cesarean section, Outcomes, Pregnancy, Adverse outcomes

## Abstract

**Background:**

Maternal age at delivery and cesarean section rates are increasing. In older women, the decision on delivery mode may be influenced by a reported increased risk of surgical interventions during labor and complications with increasing maternal age. We examined the association between maternal age and adverse outcomes in low-risk primiparous women, and the risk of adverse outcomes by delivery modes, both planned and performed (elective and emergency cesarean section, operative vaginal delivery, and unassisted vaginal delivery) in women aged ≥ 35 years.

**Methods:**

A population-based registry study was conducted using data from the Medical Birth Registry of Norway and Statistics Norway including 169,583 low-risk primiparas with singleton, cephalic labors at ≥ 37 weeks during 1999 − 2009. Outcomes studied were obstetric blood loss, maternal transfer to intensive care units, 5-min Apgar score, and neonatal complications. We adjusted for potential confounders using relative risk models and multinomial logistic regression.

**Results:**

Most adverse outcomes increased with increasing maternal age. However, the increase in absolute risks was low, except for moderate obstetric blood loss and transfer to the neonatal intensive care unit (NICU). Operative deliveries increased with increasing maternal age and in women aged ≥ 35 years, the risk of maternal complications in operative delivery increased. Neonatal adverse outcomes increased mainly in emergency operative deliveries. Moderate blood loss was three times more likely in elective and emergency cesarean section than in unassisted vaginal delivery, and twice as likely in operative vaginal delivery. Low Apgar score and neonatal complications occurred two to three times more often in emergency operative deliveries than in unassisted vaginal delivery. However, comparing outcomes after elective cesarean section and planned vaginal delivery, only moderate blood loss (higher in elective cesarean section), neonatal transfer to NICU and neonatal infections (both higher in planned vaginal delivery) differed significantly.

**Conclusions:**

Most studied adverse outcomes increased with increasing maternal age, as did operative delivery. Although emergency operative procedures were associated with an increased risk of adverse outcomes, the absolute risk difference in complications between the modes of delivery was low for the majority of outcomes studied.

**Electronic supplementary material:**

The online version of this article (doi:10.1186/s12884-016-1028-3) contains supplementary material, which is available to authorized users.

## Background

Maternal age at delivery has increased during recent decades, as has the rate of cesarean section (CS) [1]. In 2014 in Norway, the average age at first childbirth was 28.7 years, 20 % of women were 35 years or older at delivey and the CS rate was 16.5 % [2]. Advanced maternal age is associated with an increased risk of obesity [3], hypertensive diseases [4–6] and diabetes [4–6], and obstetric interventions including CS [4–10]. It is not clear whether the increase in CS is caused by medical complications increasing with maternal age, or by maternal age per se [11]. Evidence exists showing that even the rate of elective CS without medical indication increases with advancing maternal age [12]. Health providers’ perception of maternal age as a risk factor may lower the threshold for CS in advanced maternal age [11]. In older women, a decision about mode of delivery might be influenced by studies reporting an excess risk of prolonged labor [4, 5] fetal distress [4, 6, 9, 13], intrapartum CS [7, 9], and operative vaginal deliveries [9]. Operative delivery may cause complications for the mother and the child [14, 15] and is associated with an economic cost to society [16]. Most studies regarding maternal age and complications after delivery have been performed on unselected populations including high risk women [5, 6, 9]. However, most pregnant women of advanced age are healthy and of higher socio-economic status [4, 5, 9, 17]. Furthermore, most studies comparing complications of elective CS and planned vaginal delivery have not examined the influence of maternal age [18–21] but rather treated maternal age as a confounder to be adjusted for [19, 22]. The difficult consultation about mode of delivery in mothers of advanced maternal age calls for more knowledge about adverse outcomes of labor in older low-risk mothers, and possible differences in such outcomes according to mode of delivery.

We examined the risk of adverse outcomes according to maternal age among low-risk primiparous women in Norway. Further, in women aged ≥ 35 years, we studied the risk of adverse outcomes after elective CS and operative vaginal delivery, compared to unassisted vaginal delivery. Finally, with the aim of helping older mothers make an informed choice of delivery mode, we compared these adverse outcomes after elective CS and planned vaginal delivery.

## Methods

We performed a population-based study using data from the Medical Birth Registry of Norway (MBRN), linked to data from Statistics Norway by the personal identification numbers given to all residents in Norway. Based on compulsory notification, since 1967 the MBRN has registered information on all births from gestational week 16 (week 12 since 2001). The parents’ demographic data, mothers’ health before and during pregnancy, interventions and complications during pregnancy and delivery and birth outcomes, are registered prospectively using a standardized notification form. The midwife or physician attending the delivery is responsible for completing the form within seven days after delivery. Information is recorded using tick boxes and free text descriptions on the form. The classification systems used for coding free text include the 10th revision of the International Classification of Diseases, 1999 (ICD-10) [23], and the NOMESCO Classification of Surgical Procedures [24]. For information on the mother’s education level and country of origin, the data from the MBRN were linked to the National Education Database and the Country of Origin at Statistics Norway. The source population comprised all women delivering their first child (≥22 gestational weeks or ≥ 500 g) from 1 January 1999 to 31 December 2009 (*n* = 262,124).

Our aim was to study low-risk women without registered medical indications for elective CS. The MBRN contains information about maternal diseases and pregnancy complications, but not the indications for CS delivery. Based on the results of a previous study [12], we therefore excluded mothers with one or more registered medical and pregnancy complications associated with elective CS delivery (Additional file 1). The final study population included 169,583 low-risk primiparous mothers with singleton, cephalic labors at ≥ 37 gestational weeks.

### Variables

The main neonatal outcome variables were Apgar scores < 7 and < 4 at 5 min, respiratory and cerebral complications, neonatal bacterial infections, and transfer to the NICU.

Respiratory complications were identified by the tick boxes “Transitory tachypnea”, “Respiratory distress”, “Meconium aspiration”, “Use of respirator” and “Continuous positive airway pressure”, and free text coded by the ICD-10 codes P 22.1, P22.8, P22.0, and P24. Cerebral complications were identified by the tick boxes “Intracranial hemorrhage”, “Cerebral irritation”, “Cerebral depression”, and “Neonatal convulsions” and free text coded by the ICD-10 codes P10, P91.3, P91.4, P91.5, and P90. Neonatal bacterial infections were identified by the tick boxes “Bacterial infection” and “Systemic antibiotics” and free text coded by the ICD-10 codes P23.1–9 and P36. Transfer to the NICU was identified by a tick box on the birth notification form.

The main maternal outcomes were obstetric blood loss and transfer to the intensive care unit (ICU). Obstetric blood loss was categorized based on the amount, visually estimated within 24 h postpartum: moderate (500 − 1500 ml) and severe (>1500 ml or blood transfusion regardless of the amount of blood loss). The amount of blood loss was identified by tick boxes or free text on the birth notification form. Transfer of the mother to the ICU was identified by a tick box.

The explanatory variable was delivery mode grouped as elective CS, emergency CS, operative vaginal delivery (forceps and vacuum) and unassisted vaginal delivery. Deliveries not performed as elective CS (the remainder) were defined as planned vaginal deliveries. The planned vaginal delivery group therefore constitute a mixture of unassisted vaginal delivery, vacuum/forceps and emergency CS. The MBRN groups CS into the following three categories: elective, emergency or unspecified (13.6). The notifying midwife/doctor must specify whether a CS was planned in advance, and also whether it was performed as an elective or emergency CS. If the delivery mode is CS without these questions being answered, or the answers are contradictory, the CS is categorized as unspecified. Elective CS was, by definition, performed >8 h after the decision was made for surgical intervention and was otherwise classified as an emergency CS. We assigned unspecified CS with CS recorded as the start of labor to the elective CS group (4.5 % of the unspecified CS), and unspecified CS with spontaneous or induced labor to the emergency CS group (95.5 % of the unspecified CS). Delivery mode (CS, vacuum, forceps, or unassisted vaginal delivery) was identified by tick boxes on the notification form.

Maternal age at delivery was categorized as < 20, 20 − 24 (reference), 25 − 29, 30 − 34, and ≥ 35 years. Because we specifically wanted to gain knowledge of adverse outcomes in older women according to mode of delivery, we focused on women aged ≥ 35 years when assessing the relation between delivery mode and adverse outcomes.

The background variables included: the mother’s education level (high: tertiary-level education, ≥ 14 years (reference); middle: upper secondary level, 11 − 13 years; low: compulsory, < 11 years); the mother’s country of origin (Group 1 (EU/EEA, USA, Canada, Australia and New Zealand, reference) or group 2 (the remaining countries)); the year of delivery (1999 − 2000 (reference), 2001 − 03, 2004 − 06, or 2007 − 09); marital status (living with a partner (reference) or living alone); daily cigarette smoking (no (reference) or yes); and hospital size based on annual number of deliveries (<1500, 1500 − 2999 (reference), and ≥ 3000). Gestational age was based on ultrasound estimations if available, or on the last menstrual period. Major congenital malformations were coded using ICD-10 and grouped according to European Surveillance of Congenital Anomalies definitions [25].

### Statistics

The low-risk population was established using frequency analyses and contingency tables. We wanted to study women without registered indications for elective CS. Women with medical conditions significantly associated with elective CS were excluded [12] (Additional file 1). In the remaining low-risk population, the associations between maternal age and adverse obstetric and neonatal outcomes, and between modes of delivery and the adverse outcomes, were explored using descriptive statistics, contingency tables with chi-square tests, and t-tests. Similar procedures were used to evaluate confounding variables. For the first aim, log binomial regression models in the generalized linear model program were used to compute relative risks (RRs) and 95 % confidence intervals (CI). For the second aim multinomial logistic regression was used to compute relative risk ratios (RRRs) and 95 % CI. We built regression models to estimate adjusted RRs (aRR) of adverse outcomes by maternal age (reference category 20 − 24 years), and adjusted RRRs (aRRR) of adverse outcomes by modes of delivery, reference category being unassisted vaginal delivery, and, finally, adjusted RRs of adverse outcomes in elective CS compared with planned vaginal delivery (remaining deliveries). Potential confounders were recruited on the basis of previous knowledge and current literature. Factors significantly associated with maternal age, with elective CS and with adverse outcomes in bivariate analyses were kept in the model. Gestational age was modeled as a continuous factor and all other factors as categorical. To explore the direct effect of maternal age on adverse outcomes we adjusted for modes of delivery. However, adjusting for intermediate variables on the causal path may introduce bias in the case of unmeasured variables [26]; we thus present both crude and adjusted RRs. In these analyses, maternal age was modeled both as a continuous and a categorical term. Mothers with missing values for maternal age (*n* = 13) were excluded. In addition to studying low-risk women, sensitivity analyses were performed in the total population. For the neonatal outcomes, we performed sensitivity analyses restricted to women who delivered neonates without major congenital malformations registered at birth [25]. The results were considered significant at *p* < 0.05 (two-sided). The data were analyzed with SPSS version 20 (SPSS Inc., Chicago, IL, USA) and STATA version MP 13.1 (http://www.stata.com).

## Results

At gestational age ≥ 37 weeks, 69 percent of primiparas were low-risk; in women aged < 20 the corresponding rate was 71 % and in those aged ≥ 35 years it was 59 %. In this population, the overall prevalence of elective CS without registered medical indications was 1 %, increasing from 0.6 % in women aged < 20 years to 3.4 % in those ≥ 35 years (Table 1). The respective rates of emergency were 5.3 % and 16.9 %, and of assisted vaginal delivery were 10.2 % and 22.4 %.

**Table 1:** Modes of delivery by maternal age (low-risk population of primiparous women, delivering singletons in cephalic version at term (≥37gestational weeks). Norway, 19992009, *n* = 169,583)

Maternal age	Deliveries	Elective Cesarean section		Planned vaginal delivery					
				Emergency Cesarean section		Operative vaginal		Unassisted vaginal	
Deliveries (Row%)	169,583	1689 (1.0 %)		15,362 (9.0 %)		27,447 (16.2 %)		125,085 (73.9 %)	
		*n*	Row %	*n*	Row %	*n*	Row %	*n*	Row %
<20	9398	53	0.6	502	5.3	958	10.2	7885	83.9
20 − 24	43,658	264	0.6	2929	6.7	5663	13.0	34,802	79.8
25 − 29	67,338	487	0.7	5652	8.4	10,918	16.2	50,281	74.7
30 − 34	38,190	512	1.3	4421	11.6	7439	19.5	25,818	67.7
≥35	10,999	373	3.4	1858	16.9	2469	22.4	6299	57.3

### Adverse outcomes by maternal age

Table 2 shows that the proportion of adverse outcomes increased with increasing maternal age (*p*-trend < 0.001). Except for moderate blood loss (12.4 % in women aged 20 − 24 years, and 18.3 % in women aged ≥ 35 years) and neonatal transfer to the NICU (5.2 % and 7.3 %), the increases were low. Table 3 shows that maternal age was associated with increased RR of adverse outcomes. Adjustments for sociodemographic factors decreased the RRs only slightly. Further adjustments for mode of delivery further decreased the RR, and only maternal blood loss (moderate maternal blood loss aRR 1.13, 95 % CI 1.07 − 1.19), Apgar < 4 at 5 min (aRR 1.63, 95 % CI 1.17 − 2.26) and neonatal infections (aRR 1.29, 1.14 − 2.15) remained significant when comparing women aged ≥ 35 years with those aged 20 − 24 years. Maternal blood loss increased with increasing maternal age independently of delivery mode, but neonatal complications increased with maternal age only in unassisted vaginal delivery.

**Table 2:** Maternal and neonatal adverse outcomes by maternal age. Percentages and 95 % confidence intervals (CI) shown for a low-risk population of primiparous women, delivering singletons in cephalic version at term (≥37gestational weeks). Norway, 1999 − 2009, *n* = 169,583

Maternal adverse outcomes						Neonatal adverse outcomes					
			Blood	Blood	ICU	Apgar	Apgar	Respiratory	Cerebral	Infections	NICU
			loss	loss		<7, 5 min	<4, 5 min				
			500−	>1500							
			1500 ml	ml							
Deliveries, *n*			24,250	1925	327	2200	542	2876	1101	4070	9624
Overall (%)			(14.3 %)	(1.1 %)	(0.2 %)	(1.3 %)	(0.3 %)	(1.7 %)	(0.6 %)	(2.4 %)	(5.7 %)
			% (95 % CI)	% (95 % CI)	% (95 % CI)	% (95 % CI)	% (95 % CI)	% (95 % CI)	% (95 % CI)	% (95 % CI)	% (95 % CI)
<20	9398	5.5	10.6 (10.0-11.2)	0.8 (0.6-1.0)	0.1 (0.1-0.3)	1.1 (0.9-1.3)	0.3 (0.2-0.4)	1.2 (1.0-1.4)	0.5 (0.3-0.6)	1.5 (1.2-1.7)	4.7 (4.3-5.1)
20 − 24	43,658	25.7	12.4 (12.1-12.7)	1.0 (0.9-1.0)	0.1 (0.1-0.2)	1.2 (1.1-1.3)	0.3 (0.2-0.3)	1.5 (1.4-1.6)	0.6 (0.5-0.6)	2.1 (1.9-2.2)	5.2 (5.0-5.4)
25 − 29	67,338	39.7	14.3 (14.1-14.6)	1.1 (1.0-1.2)	0.2 (0.2-0.2)	1.3 (1.2-1.3)	0.3 (0.3-0.4)	1.7 (1.6-1.8)	0.6 (0.6-0.7)	2.3 (2.2-2.4)	5.6 (5.4-5.7)
30 − 34	38,190	22.5	16.2 (15.8-16.6)	1.3 (1.2-1.5)	0.3 (0.2-0.3)	1.4 (1.3-1.6)	0.3 (0.3-0.4)	1.9 (1.8-2.0)	0.7 (0.6-0.8)	1.8 (2.6-3.0)	6.3 (6.1-6.6)
≥35	10,999	6.5	18.3 (17.5-19.0)	1.8 (1.6-2.1)	0.2 (0.2-0.3)	1.8 (1.6-2.1)	0.5 (0.4-0.7)	2.2 (1.9-2.5)	1.0 (0.9-1.2)	3.6 (3.3-4.0)	7.3 (6.8-7.8)
*p*-trend^a^											

*ICU* Intensive care unit, *NICU* Neonatal intensive care unit^a^ *p*-trend < 0.001 for all groups

**Table 3:** Crude and adjusted relative risks (RR) with 95 % confidence interval (CI) for adverse outcomes by maternal age in a low-risk population of primiparous women delivering singletons in cephalic version at term (≥37 gestational weeks). Norway, 1999 − 2009, *n* = 169,583

Maternal age (years)	<20		20 − 24	25 − 29		30 − 34		≥35	
Deliveries (*n*, %)	(9398; 5.5 %)		(43,658; 25.7 %)	(67,338; 39.7 %)		(38,190; 22.5 %)		(9615; 5.7 %)	
	RRc (95 % CI)	RRadj (95 % CI)^a^		RRc (95 % CI)	RRadj (95 % CI)^a^	RRc (95 % CI)	RRadj(95 % CI)^a^	RRadj (95 % CI)	RRadj (95 % CI)^a^
Maternal complications									
Blood loss 500 − 1500 ml	0.86 (0.80-0.92)	0.91 (0.85-0.98)	1	1.16 (1.12-1.20)	1.09 (1.05-1.13)	1.31 (1.26-1.36)	1.12 (1.08-1.17)	1.48 (1.40-1.56)	1.13 (1.07-1.19)
Blood loss ≥ 1500 ml	0.82 (0.64-1.05)	0.85 (0.66-1.09)	1	1.14 (1.01-1.29)	1.09 (0.97-1.23)	1.40 (1.23-1.59)	1.24 (1.08-1.41)	1.89 (1.60-2.24)	1.49 (1.25-1.77)
Transfer to ICU	1.03 (0.58-1.84)	1.02 (0.56-1.86)	1	1.30 (0.96-1.75)	1.26 (0.93-1.71)	1.78 (1.30-2.44)	1.54 (1.12-2.13)	1.64 (1.01-2.59)	1.17 (0.73-1.86)
Neonatal complications									
Apgar < 7 at 5 min	0.91 (0.73-1.12)	0.97 (0.78-1.21)	1	1.08 (0.97-1.20)	1.00 (0.90-1.12)	1.23 (1.09-1.39)	1.01 (0.89-1.14)	1.58 (1.34-1.86)	1.12 (0.95-1.33)
Apgar < 4 at 5 min	1.07 (0.70-1.64)	1.09 (0.71-1.69)	1	1.23 (0.98-1.55)	1.21 (0.96-1.52)	1.32 (1.02-1.69)	1.18 (0.91-1.52)	2.04 (1.48-2.80)	1.63 (1.17-2.26)
NICU	0.91 (0.82-1.00)	0.97 (0.87-1.07)	1	1.08 (1.02-1.13)	0.98 (0.93-1.04)	1.23 (1.16-1.30)	1.02 (0.96-1.08)	1.41 (1.30-1.52)	1.05 (0.97-1.14)
Respiratory	0.80 (0.65-0.98)	0.87 (0.71-1.06)	1	1.13 (1.03-1.24)	1.03 (0.94-1.14)	1.26 (1.13-1.40)	1.04 (0.93-1.15)	1.44 (1.24-1.67)	1.06 (0.91-1.23)
Cerebral	0.84 (0.61-1.16)	0.95 (0.69-1.31)	1	1.11 (0.95-1.29)	0.97 (0.82-1.13)	1.25 (1.05-1.48)	0.94 (0.79-1.12)	1.82 (1.46-2.28)	1.20 (0.96-1.51)
Infection	0.71 (0.59-0.85)	0.78 (0.65-0.93)	1	1.12 (1.04-1.22)	1.03 (0.95-1.12)	1.35 (1.23-1.47)	1.10 (1.01-1.21)	1.76 (1.57-1.98)	1.29 (1.14-2.15)

*RRc* crude relative risk, *RRadj* adjusted relative risk, *ICU* Intensive care unit, *NICU* Neonatal intensive care unit;^a^ Adjusted for year of delivery, country, hospital, elective caesarean section, gestational age

### Adverse outcomes by mode of delivery

For our second aim, we analyzed women ≥ 35 years at delivery (Table 4). The table shows the RRRs of adverse outcomes after elective CS, emergency CS and operative vaginal delivery, compared with unassisted vaginal delivery. The risk of moderate blood loss was twice as high in operative vaginal delivery, and three times as high in CS, compared with unassisted vaginal delivery. The overall absolute risk of severe blood loss was low (1.8 %), but was 70 % higher in operative compared with unassisted vaginal delivery. Only 26 (0.2 %) of mothers were transferred to ICUs, but the risk increased after operative vaginal delivery compared with unassisted vaginal delivery.

**Table 4:** Adverse maternal and neonatal outcomes in women with unassisted vaginal delivery, elective cesarean section (CS), emergency cesarean section (CS) and operative vaginal delivery

		unassisted vaginal delivery	Elective CS	Emergency CS	Operative vaginal delivery	Elective CS		Emergency CS		Operative vaginal delivery	
		*n* (%)	*n* (%)	*n* (%)	*n* (%)	RRR_c_ (95 % CI)	RRR_adj_^a^ (95 % CI)	RRR_c_ (95 % CI)	RRR_adj_^a^ (95 % CI)	RRR_c_ (95 % CI)	RRR_adj_^a^ (95 % CI)
		6299 (57.3.0)	373 (3.4)	1858 (16.9)	2469 (22.4)						
PPH 500-1500	2008	764 (12.1)	103 (27.6)	591 (31.8)	550 (22.3)	2.76 (2.18-3.51)	2.97 (2.31-3.83)	3.38 (2.99-3.82)	3.23 (2.84-3.67)	2.08 (1.84-2.34)	2.01 (1.77-.2.27)
PPH > 1500 ml	198	90 (1.4)	8 (2.1)	35 (1.9)	65 (2.6)	1.51 (0.73-3.14)	1.63 (0.75-3.55)	1.32 (0.89-1.96)	1.16 (0.77-1.74)	1.87 (1.35-2.58)	1.73 (1.25-2.41)
ICU	26	7 (0.1)	1 (0.3)	6 (0.3)	12 (0.5)	2.42 (0.30-19.69)	1.13 (0.12-11.05)	2.91 (0.98-8.68)	2.62 (0.84-8.14)	4.39 (1.73-11.16)	4.26 (1.65-10.96)
Apgar < 7	202	64 (1.0)	1 (0.3)	66 (3.6)	71 (2.9)	0.26 (0.04-1.89)	0.28 (0.04-2.06)	3.59 (2.53-5.08)	3.69 (2.57-5.31)	2.88 (2.05-4.06)	2.94 (2.08-4.17)
Apgar < 4	58	24 (0.4)	0	14 (0.8)	20 (0.8)	0.00		1.98 (1.02-3.84)	2.30 (1.14-4.62)	2.13 (1.18-3.87)	2.41 (1.30-4.47)
NICU	796	282 (4.5)	16 (4.3)	249 (13.4)	249 (10.1)	0.96 (0.58-1.61)	0.86 (0.50-1.46)	3.30 (2.76-3.95)	3.40 (2.82-4.10)	2.40 (2.01-2.86)	2.45 (2.05-2.94)
Respiratory	238	82 (1.3)	5 (1.3)	79 (4.3)	72 (2.9)	1.03 (0.42-2.56)	0.94 (0.36-2.46)	3.37 (2.46-4.61)	3.59 (2.59-4.97)	2.27 (1.65-3.14)	2.35 (1.70-3.25)
Cerebral	114	34 (0.5)	1 (0.3)	37 (2.0)	42 (1.7)	0.50 (0.07-3.63)	0.50 (0.06-3.86)	3.74 (2.34-5.98)	3.52 (2.17-5.71)	3.19 (2.02-5.02)	3.00 (1.89-4.76)
Infection	401	154 (2.4)	4 (1.1)	121 (6.5)	122 (4.9)	0.43 (0.16-1.17)	0.43 (0.16-1.19)	2.78 (2.18-3.55)	2.92 (2.27-3.77)	2.07 (1.63-2.64)	2.13 (1.67-2.73)

*RRRc* crude relative risk ratio, *RRRadj* adjusted relative risk ratio, *ICU* Intensive care unit, *NICU* Neonatal intensive care unit

Unassisted vaginal delivery is the reference mode of delivery (primipara ≥ 35 years, low-risk, ≥ 37 weeks of gestation, 19999 − 2009, *n* = 10,999)

^a^Adjusted for year of delivery, country, hospital size, gestational age and maternal age

Most of the newborns were healthy and had no complications. There was no significant difference in adverse neonatal outcomes between elective CS and unassisted vaginal delivery, but the risks were between two and three times higher in emergency CS or operative vaginal deliveries compared with unassisted vaginal deliveries. Neonatal infection was the most common neonatal complication and the risk was more than twice as high in emergency CS (6.5 %, aRRR 2.92, 95 % CI 2.27 − 3.77) and operative vaginal deliveries (4.9 %, aRRR 2.13, 95 % CI 1.67 − 2.73) compared with unassisted vaginal deliveries (2.4 %).

Since some congenital anomalies are more frequent in offspring born to older women, we performed a sensitivity analysis where we excluded women whose infants were diagnosed with major congenital anomalies after delivery. The absolute risks of adverse neonatal complications were then slightly lower. However, the aRRRs of adverse outcome according to delivery mode (reference being unassisted vaginal delivery) were not substantially different in this population. Finally, to explore whether our study could inform the choice of delivery mode for older mothers, we compared outcomes after elective CS with planned vaginal delivery (emergency CS, operative vaginal delivery and unassisted vaginal delivery). Only three of the adverse maternal and neonatal outcomes differed significantly between the two delivery modes; Adjusted RR of moderate blood loss was 1.83 (95 % CI 1.49 − 2.26), aRR of neonatal infection was 0.31 (95 % CI 0.12 − 0.84), and aRR for infant’s transfer to the NICU was 0.61 (95 % CI 0.37 − 0.99). For the other outcomes the estimates did not differ significantly (Table 5).

**Table 5:** Risk difference and relative risk (RR) with 95 % confidence interval (CI) of adverse maternal and neonatal outcomes after elective caesarean section (CS) compared with planned vaginal delivery (remaining deliveries). Shown for a low-risk population of primiparous women ≥ 35 years when delivering singletons in cephalic version at term (≥37 gestational weeks) Norway, 1999 − 2009, *n* = 10,999

Elective CS								
Yes				No				
Deliveries			373	10,626				
	*n*	%	%	%	Risk difference %	*P*-value^a^	RR_c_ (95 % CI)	RR_adj_^b^ (95 % CI)
Maternal complications								
Blood loss 500 − 1500 ml	2008	18.3	27.6	17.9	9.7	0.000	1.54 (1.30-1.82)	1.83 (1.49-2.26)
Blood loss ≥ 1500 ml	198	1.8	2.1	1.8	0.3	0.611	1.20 (0.60-2.42)	1.43 (0.69-2.93)
ICU	26	0.2	0.3	0.2	0.1	0.898	1.14 (0.15-8.39)	0.69 (0.08-5.58)
Neonatal complications								
Apgar < 7 at 5 min	202	1.8	0.3	1.9	-1.6	0.025	0.14 (0.02-1.01)	0.17 (0.02-1.20)
Apgar < 4 at 5 min	58	0.5	0.0	0.5	-0.5	0.153		
NICU	796	7.3	4.3	7.4	-3.1	0.020	0.59 (0.36-0.96)	0.61 (0.37-0.99)
Respiratory	238	2.2	1.3	2.2		0.266	0.61 (0.25-1.47)	0.70 (0.29-1.70)
Cerebral	114	1.0	0.3	1.1	-0.9	0.136	0.25 (0.04-1.80)	0.38 (0.05-2.78)
Infection	401	3.6	1.1	3.7	-2.6	0.007	0.29 (0.11-0.76	0.31 (0.12-0.84)

*ICU* Intensive care unit, *NICU* Neonatal intensive care unit, *RRc* crude relative risk, *RRadj* adjusted relative risk

^a^un-adjusted Chi2; ^b^Adjusted for year of delivery, country of origin, hospital size, gestational age and maternal age

## Discussion

We found an increase in maternal and neonatal adverse outcomes by increasing maternal age (*p*-trend < 0.001). However, when adjusting for modes of delivery, only the increase in maternal blood loss, Apgar score < 4 at 5 min, and neonatal infections remained statistically significant. In women aged ≥ 35 years at delivery, maternal blood loss was more frequent in operative deliveries (especially emergency CS) than in unassisted vaginal deliveries. The risk of neonatal complications was substantially increased after the emergency operative procedures during labor compared to unassisted vaginal delivery. However, when comparing elective CS to planned vaginal delivery (remaining deliveries), only moderate blood loss, transfer to NICU and neonatal infections differed between the planned modes, and the neonatal risks were lower in elective CS.

### Strengths and limitations

The current study was based on data from the nationwide MBRN with compulsory notification of all births in the country; errors due to selection bias are therefore unlikely. Validation studies on information about maternal disease in the MBRN have shown satisfactory results [27–29]. However, there may be underreporting of maternal medical conditions.

There are limitations to this study. The low-risk population was established by excluding women with complications associated with elective CS as a proxy for medical indications for elective CS. Indications for operative deliveries are not well registered in the MBRN, whereas pregnancy and delivery complications are. However, as surveillance and notification of medical complications to the MBRN may have been more vigilant in older than in younger women, we may have excluded a higher proportion of older women when constructing the low-risk population. This may attenuate the estimates of any associations between maternal age and adverse outcomes. Unfortunately, we did not have access to information on whether the CS deliveries were booked in advance, only how they were performed. We therefore used elective CS as a proxy for planned CS and the remainder as planned vaginal delivery. The clinical assessment of maternal blood loss [30] and Apgar score [31] may suffer from subjectivity. The assessment of blood loss is more accurate in CS by recording the content of drainage bottles. In vaginal delivery, the blood loss is mainly visually assessed and large blood loss tends to be underestimated [30]. Our effect estimates of blood loss in vaginal deliveries may therefore be underestimated. Transfer to the NICU does not include information about the time spent in the unit, and in some cases shorter stay may be related to practical routines differing between delivery units rather to neonatal morbidity. Associations between body mass index (BMI) and maternal age [3], delivery modes, low Apgar scores, and obstetric blood loss, have been described [32]. Information on maternal height and weight was unfortunately not available in the MBRN, and we were therefore unable to adjust for a possible confounding by BMI. Although our sample is large, the limited number of women in the age group above 35 years and the rarity of some of the adverse outcomes resulted in a limited power for some of the estimations.

### Interpretation

#### Adverse outcomes by maternal age

We found that the adverse outcomes increased with maternal age. Obstetric blood loss 500 − 1500 ml showed the greatest absolute increase with age, from 12 % in women aged 20 − 24 years to 18%in women aged ≥ 35 years. This finding is in accordance with Luke et al. who reported increased risk of obstetric blood loss in advanced maternal age in an unselected population including high-risk groups [5]. The decrease in myometrial contractility and reduced effect of oxytocin in older mothers [33] could play a role. We found increased risk of maternal transfer to the ICU in the higher age groups, in line with existing evidence of increased risk of major obstetric blood loss, hypertensive disorders of pregnancy and sepsis in advanced maternal age [34].

In our study, the risk of infant transfer to the NICU increased from 5 % in women aged 20 − 24 years to 7 % in those aged ≥ 35 years. Klemetti et el. found an increase from 11 % to 21 % across the same age range in a Finnish population study [9]. A true increased risk of neonatal morbidity with increasing maternal age is possible, but a lower threshold for transfer to NICU in older mothers could also be a factor [35]. Unfortunately, information on umbilical pH was not available in the registry. However, several studies have found that low Apgar scores at 5 min do predict neonatal morbidity [36, 37]. In line with our results, Klemetti et al. found an increase in low Apgar scores (<7 at 5 min) with increasing maternal age [9], and this is further supported by a study from the United States [6]. Others have shown an increased risk of fetal distress [4] and meconium stained amniotic fluid [38] with increasing maternal age, in which impaired placental blood flow may play a role [39]. In our study, the increased risk of neonatal bacterial infections with increasing maternal age may partly be explained by the increased risk of prelabor rupture of membranes [5, 40] and dystocia seen in older women [4, 5]. Supported by an Italian study from 1999 [41], and a more recent Danish study [19] we found that respiratory complications increased with maternal age. In a Swedish registry study, Ekeus et al. found an age related increased risk of neonatal convulsions and neonatal encephalopathy, in women delivered at term by both unassisted vaginal, vacuum extraction and emergency CS, and of intracranial hemorrhage after unassisted vaginal delivery [42]. Based on our observational study we cannot draw conclusions about causal relations. However, the gradual increase in adverse outcomes by maternal age, also supported by other studies, may suggest a real age effect [43].

### Adverse outcomes by mode of delivery

We found an increasing risk of operative deliveries with increasing maternal age. Of women aged ≥ 35 years, 3 % were delivered by elective CS, 22 % by emergency CS and 17 % by operative vaginal delivery. The risk of adverse outcomes was higher in operative deliveries, especially emergency operative deliveries, than in unassisted vaginal delivery. However, comparing elective CS with planned vaginal (remaining) deliveries, only moderate maternal blood loss (twice the risk in elective CS), infant transfer to the NICU and neonatal infections (both lower risk in elective CS) differed significantly between the planned delivery modes. The overall proportion of moderate blood loss was 18 % in women aged 35 years and older. We found this outcome to be three times more frequent in elective CS relative unassisted vaginal delivery, in accordance with the findings of Karlstrøm et al. [44]. Our results were also in agreement for obstetric blood loss associated with emergency CS. In contrast to our study, Allen et al. reported decreased risk of early postpartum blood loss in CS without labor compared with the remaining deliveries [18, 45]. In our study, the risk of severe blood loss/blood transfusion increased by 70 % in operative vaginal delivery compared with unassisted vaginal delivery. This contrasts with both a Danish retrospective population study [46] and an Australian retrospective hospital study [47]. With regard to maternal transfer to ICUs, we found a substantially increased risk after operative vaginal versus unassisted vaginal delivery (aRRR 4.26, 95 % CI 1.65 − 10.96), but not after elective of emergency CS. However, the numbers were small and the confidence intervals wide. A large study from Canada comparing elective CS for breech delivery and planned vaginal delivery in low-risk women reported increased risk of serious maternal complications, including serious obstetric blood loss and sepsis, after elective CS [22].

We did not find a difference in risk of adverse complications between elective CS and unassisted vaginal delivery, but this may be because of a lack of power. Conversely, the risk of low Apgar score and neonatal transfer to the NICU was more than doubled after emergency CS and operative vaginal delivery compared with unassisted vaginal delivery. Karlstrøm et al. found a three-fold increased risk of low Apgar score after emergency CS and half the risk after elective CS compared with vaginal deliveries [44]. Other studies have found an increased risk of neonatal transfer to the NICU after elective CS compared with planned vaginal delivery [20, 48]. However, these studies were performed in populations where most women were aged < 35 years. In the current study of women ≥ 35 years, those deliveries that were not performed as elective CS had substantially greater risk of emergency operative interventions, increasing the risk of respiratory distress and other complications that may lead to transfer to the NICU. Furthermore, older mothers have increased risk of premature rupture of membranes [5], dystocia [4, 5], and fetal distress [4]. In women aged ≥ 35, we found twice the risk of neonatal respiratory complications after emergency CS and operative vaginal delivery compared to unassisted vaginal delivery. We did not find significant differences between elective CS and planned vaginal delivery. This is in contrast to other studies reporting higher risk of respiratory complications after elective CS compared to planned vaginal deliveries [19, 20, 49]. Delivery by CS before labor, without the stress of vaginal labor involving thoracic compression [50] and a surge of catecholamines [51] is associated with respiratory depression. The increased risk of immaturity associated with an elective CS before 39 gestational weeks may add to the increased risk [19]. However, in older mothers, dystocia, fetal distress and birth trauma associated with emergency operative delivery may contribute to an increase in respiratory complications after planned vaginal deliveries [52, 53]. We found that cerebral complications were twice as high after emergency CS and three times as high after operative vaginal delivery compared to unassisted vaginal delivery. In line with this finding, a previous study from the Netherlands of 53 full-term neonates with diagnosed intracranial hemorrhage found that most were delivered by operative vaginal delivery and only 26 % by unassisted vaginal delivery [54]. We found twice the risk of neonatal infection in emergency CS compared with unassisted vaginal delivery, supported by Karlstrøm et al. [44]. In Norway, only recently, induction of labor is recommended after 24 h of prelabor rupture of membranes [55], and this may lead to reduced neonatal infection.

## Conclusion

We found an increase in maternal and neonatal adverse outcomes with increasing maternal age, as well as an increase in operative deliveries. In low-risk primiparous women aged 35 years and older, operative delivery increased the risk of maternal blood loss. Emergency operative procedures during labor substantially increased the risk of all neonatal complications studied. However, comparing elective CS with planned vaginal delivery, only transferal to the NICU and neonatal infection differed significantly, and most of the infants were healthy regardless of delivery mode. In accordance with existing guidelines [55, 56], we support the encouragement of planned vaginal delivery in older mothers.

## Additional file

Additional file 1: Women with the following medical conditions were excluded from the total population (primiparous women, gestational age ≥ 22 weeks or birth weight ≥ 500 g, *n* = 262,124 pregnancies, *n* = 267,373 births) to establish the low-risk population (*n* = 169,583). (DOC 56 kb)

## Abbreviations

CI: Confidence interval
CS: Cesarean section
ICU: Intensive care unit
MBRN: Medical birth registry of Norway
NICU: Neonatal intensive care unit
RR: Relative risk
RRR: Relative risk ratio
