# Supplementary material for: Elective cesarean section or not? Maternal age and risk of adverse outcomes at term: a population-based registry study of low-risk primiparous women
Source: BMC Pregnancy Childbirth. 2016 Aug 17;16:230. doi: 10.1186/s12884-016-1028-3 (PMC4988032; doi:10.1186/s12884-016-1028-3)
Supplement: Additional file 1: — Women with the following medical conditions were excluded from the total population (primiparous women, gestational age ≥ 22 weeks or birth weight ≥ 500 g, n = 262,124 pregnancies, n = 267,373 births) to establish the low-risk population (n = 169,583). (DOC 56 kb) [file 12884_2016_1028_MOESM1_ESM.doc]

Additional file 1 Women with the following medical conditions were excluded from the total population (primiparous women, gestational age ≥ 22 weeks or birth weight ≥ 500 grams, n = 262,124 pregnancies, n = 267,373 births) to establish the low-risk population (*n* = 169,583).

|  | Deliveries | | |
| --- | --- | --- | --- |
| **Medical conditions of the women ^a^** |  |  |  |
|  | n |  | % |
| Previous myomectomia | 216 |  | 0.1 |
| Chronic hypertension | 1275 |  | 0.5 |
| Cardiac disease | 1697 |  | 0.6 |
| Kidney diseases | 1843 |  | 0.7 |
| Thyroid disease | 3319 |  | 1.3 |
| Diabetes before pregnancy | 1946 |  | 0.7 |
| Asthma | 12,126 |  | 4.6 |
| Neurologic diseases | 2282 |  | 1.1 |
| Inflamm Rheumatic diseases | 1378 |  | 0.5 |
| Inflammatory bowel disease | 1080 |  | 0.4 |
| Cancer | 513 |  | 0.2 |
| ART ^b^ | 8456 |  | 3.2 |
| Mental conditions |  |  |  |
| Fear of delivery | 2256 |  | 0.9 |
| Other | 6602 |  | 2.5 |
| Gestational diabetes | 2158 |  | 0.8 |
| Hypertension without preeclampsia | 5705 |  | 2.2 |
| Preeclampsia | 15,104 |  | 5.8 |
| Placenta praevia | 584 |  | 0.2 |
| Herpes genitalis | 282 |  | 0.1 |
|  |  |  |  |
|  | Births | | |
| **Medical conditions of the fetus ^a^** |  |  |  |
|  | n |  | % |
| Plurality | 10,444 |  | 3.9 |
| Gestational age < 37 weeks | 22,330 |  | 8.4 |
| IUGR ^c^ | 10,247 |  | 3.8 |
| Birthweight ≥ 4500 g | 6748 |  | 2.5 |
| Breech | 15,191 |  | 5.7 |
| Transverse lie | 798 |  | 0.3 |
| Oligohydramnion | 317 |  | 0.1 |
| Polyhydramnion | 105 |  | 0.0 |
| Congenital malformations ^d^ | 671 |  | 0.3 |
| ^a^ A woman or child may have more than one condition ^b^ Assisted Reproductive Techniques ^c^ Intrauterine growth restriction (small for gestational age (SGA) 2.5% or ICD-10 O365) ^d^ Prenatal diagnosis in pregnancies lasting more than 21 weeks. | | | |
|  |  |  |  |
|  |  |  |  |
